# *Mangifera Indica* leaf extracts promote hair growth via activation of Wnt signaling pathway in human dermal papilla cells

**DOI:** 10.1080/19768354.2022.2085790

**Published:** 2022-06-11

**Authors:** Haesoo Jung, Da-Min Jung, Sang-Soo Lee, Eun-Mi Kim, Kyungah Yoon, Kee K. Kim

**Affiliations:** a Department of Biochemistry, College of Natural Sciences, Chungnam National University, Daejeon, Republic of Korea; b Department of Predictive Toxicology, Korea Institute of Toxicology, Daejeon, Republic of Korea; c Department of Clinical Laboratory Science, Daejeon Health Institute of Technology, Daejeon, Republic of Korea

**Keywords:** Hair loss, *Mangifera indica* leaf extract, Wnt signaling pathway, *DKK1*, SRD5A2

## Abstract

The crosstalk between androgens and Wnt signaling pathways is critical in the hair growth cycle. Therefore, natural products that target these two pathways for the inhibition of hair loss are sought after. In this study, we investigated the effect of water extracts of *Mangifera indica* leaves (WEML) on hair growth. WEML treatment significantly reduced the expression levels of both dickkopf-1 (*DKK1*) and type 2 5α-reductase (SRD5A2) involved in Wnt signal suppression activity and dihydrotestosterone (DHT) synthesis, respectively, in human follicle dermal papilla cells (HFDP). In addition, WEML treatment effectively upregulated Wnt target genes and downregulated *DKK*1 expression that was increased by DHT treatment. Degranulation analysis in rat basophilic leukemia mast cell line (RBL-2H3) using β-hexosaminidase release assay confirmed that WEML did not exhibit allergenic activity. Furthermore, hair growth was significantly enhanced in *in vivo* mice model treated with WEML. These results suggest that *M. indica* leave extract contains bioactive materials that can be used to treat hair loss.

## Introduction

The hair growth process involves a cycle of a growth phase called anagen, a transition phase catagen, and a resting phase telogen (Alonso and Fuchs 2006). Disorders caused by the interruption of this normal cycle of hair production are referred to as hair loss or alopecia (Malkud 2015; Pratt et al. 2017). Hair loss may be caused by pregnancy, genetic factors, hormone secretion abnormalities, aging, extreme diet, mental stress, irregular life, febrile disease, and excessive use of chemical products. In addition to congenital factors, hair loss increases because of acquired factors, such as various environmental stresses. Previously, hair loss was considered a problem affecting the middle-aged population alone; however, an increase in hair loss is observed in young individuals at present, and the severity is high enough to be classified as a disease (Tamashunas and Bergfeld 2021). As the global interest in preventing and treating hair loss increases, studies on therapeutic agents and hair growth-promoting substances for hair loss suppression have also increased. The search for functional materials derived from natural products is continuously increasing.

Dermal papilla cells secrete various signal transmitters that regulate the growth cycle of hair follicles and play an important role in hair growth (Lachgar et al. 1996). Testosterone, a steroid sex hormone of the androgen group secreted from the testis into the blood is converted to dihydrotestosterone (DHT) by SRD5A2. DHT has a higher binding affinity for androgen receptor (AR) than that of testosterone, and this high binding affinity induces strong transcriptional activity of AR (Thornton et al. 1993; Mohler et al. 2011; Choi 2020). The increase in transcriptional activity of AR induced by DHT increases the expression of dickkopf-1 (*DKK1*), a specific inhibitor of Wnt coreceptors in the Wnt/β-catenin pathway. Dysregulation of the Wnt/β-catenin signaling pathway by *DKK1* affects particular processes, such as hair growth. Finasteride, the most effective chemical drug available at present for treating hair loss, inhibits the conversion of testosterone to DHT by inhibiting the activity of SRD5A2, thereby inducing hair growth and slowing the progression of hair loss (Kaufman and Dawber 1999; Finn et al. 2006). However, finasteride treatment has side effects such as allergic contact dermatitis, decreased libido, gynecomastia in men, decreased ejaculation, and presents a fatal risk in women of childbearing age (Irwig and Kolukula 2011; Guo et al. 2016). Therefore, physiologically active substances derived from natural products, which show few side effects in the prevention and treatment of hair loss, are sought after.

*Mangifera indica* (mango) is a perennial tropical fruit plant belonging to the Anacardiaceae family and is one of the varieties of mangoes with an apple-like shape (Shah et al. 2010; Noratto et al. 2010). The dicotyledonous plant has long spear-shaped leaves that are 30 cm in length. The plant contains mangiferin, phenols and various constituents, including glucose, flavonoids, arabinose, xylose, rhamnose, galactose, leucine, tyrosine, valine, protocatechuic acid, alanine, glycine, tannins, kinic acid, α-pinene, and β-pinene (VI). Among the components of *M. indica* extract, the polyphenol mangiferin is known to show various bioactivities such as anti-inflammatory, anti-diabetic, immunomodulatory, anti-tumor, and antioxidant activities. It has been reported that anthocyanins present in *M. indica* leaves have positive effects on diabetic retinopathy and diabetic vasculopathy when continuously ingested (Itoh et al. 2017; Ngo et al. 2019). The various physiological activities of *M. indica* leaves and the advantages of natural substances are expected to have a positive effect on hair loss.

Our previous study showed that *M. indica* leaves contain bioactive properties; however, their effects on hair growth, such as in the inhibition of hair loss-related gene expression, are still unknow (Choi et al. 2018). In this study, the effects of a water extract of *M. indica* leaves (WEML) on the crosstalk between AR and Wnt/β-catenin signaling pathways related to the hair growth cycle were investigated in human dermal papilla cells. Furthermore, the effect of WEML on hair growth was investigated in an *in vivo* mouse model.

## Materials and methods

### Animals

Male C57BL/6J mice (age, 8 weeks) purchased from Daehan Biolink (Eumseong, Korea) were housed under controlled conditions (22 ± 2°C, relative humidity of 50 ± 5%, and 12 h light/dark cycle), and were provided ad libitum access to standard rodent chow (Daehan Biolink) and water. All mice were acclimated for 7 d before the experiments. All animal experiments were approved by the Animal Experimental Ethics Committee of Chungnam National University (Daejeon, Korea) and were performed in accordance with institutional guidelines. To determine the hair growth-promoting effect, the hair on the back of the mice was first removed using an animal hair clipper without damaging the skin, and then the fine hair remaining on the skin was removed using a bit hair removal cream (Church & Dwight, Kent., UK). After a one-day recovery period, 24.75 mL of distilled water, 3.75 mL of 100% ethanol, and 1.5 mL of jojoba oil (Sigma-Aldrich, St. Louis, MO, USA), which served as a moisturizing agent, were mixed and used as a control. In the control group, only the vehicle (jojoba oil only), 1% WEML, and 0.3% minoxidil (TAE GUK PHARM., Chungcheongnam-do, Korea) were added to the vehicle at different concentrations, and 200 μL of each sample was applied to the back of depilated mice using a brush once a day.

### M. indica leaf extract preparation and yield

*M. indica* leaves were collected from farms in Geumsan, Korea, in January 2021. The plant was authenticated by Professor Dr. Kee K. Kim and deposited in the Department of Biochemistry, Chungnam National University, Daejeon, South Korea. The leaves were washed with water and dried, and 100 g of the leaves was added to a reflux extractor containing 2000 mL of primary distilled water, following extraction via heating for 2 h from the boiling point of the broth. Subsequently, the extract was filtered under reduced pressure through a filter paper (Advantec No.2, Japan) to obtain a concentrated solution using a rotary vacuum evaporator. The concentrated solution was then dried using a freeze dryer, and the powder was used as a sample. The freeze-dried extract weighed 11 g, and the yield was 11%.

### Cell culture, reagents, and 3-(4,5-dimethylthiazol-2-yl)-5-(3-carboxyphenyl)-2-(4-sulfophenyl)-2H-tetrazolium, inner salt (MTS) assay

Human follicle dermal papilla (HFDP) cells were purchased from PromoCell (cat# C-12072; Heidelberg, Germany) and cultured in follicular dermal papilla cell growth medium (C-26051; PromoCell) according to the manufacturer’s protocol at 37°C in a humidified atmosphere of 5% CO_2_. The rat basophilic leukemia mast cell line RBL-2H3 cells were purchased from American Type Culture Collection (ATCC, VA, USA). RBL-2H3 cells were cultured in DMEM supplemented with 10% heat-inactivated fetal bovine serum (FBS, WELGENE) and 1% penicillin and streptomycin at 37°C in a humidified atmosphere of 5% CO_2_. DHT and Compound 48/80 were purchased from Sigma-Aldrich (St. Louis, MO, USA). To measure the mitochondrial activity of HFDP cells, cells were cultured in 96-well plates and treated with WEML. After 24 h, 20 μL MTS solution (Promega, Madison, WI, USA) was added and cells were incubated at 37°C for 1 h (Kim et al. 2020). Absorbance was measured at 490 nm using a microplate reader (ABS Plus; Molecular Devices, San Jose, CA, USA). Mitochondrial activity was measured using the following equation:

$$Mitochondrial\;activity(\text{\%})=(\frac{A_{experimental\;group}-A_{sample\;blank}}{A_{control\;group}})\times 100$$

where *A* is the absorbance for the given wavelength.

### RNA preparation and quantitative reverse transcription-polymerase chain reaction (qRT-PCR) analysis

Total RNA was extracted from HFDP cells using a Hybrid-R™ Kit (GeneAll, Seoul, Korea) according to the manufacturer’s instructions (Ling et al. 2022). cDNA was synthesized using M-MLV reverse transcriptase (Promega) and random hexamers. Primers were designed to investigate changes in hair loss-related and Wnt target genes (Supplementary table 1), and quantitative PCR (qRT-PCR) was performed. Briefly, synthesized cDNA, 2× Prime Q-master mix (Genet Bio, Nonsan-si, Korea), and 10 pmol forward and reverse primers were mixed and subjected to qRT-PCR using AriaMx (Agilent, Santa Clara, CA, USA). The cycling program was as follows: 40 cycles of 95°C for 20 s, 58°C for 20 s, and 72°C for 20 s. The specificity of each PCR product was confirmed using melting curve analysis. β-Actin was used as the internal standard.

### β-Hexosaminidase release assay

RBL-2H3 cells were seeded at a density of 5 × 10^4^ cells/well in a 96-well plate and incubated for 24 h (Ju and Kim 2010). The medium was aspirated, and cells were washed twice with siraganian buffer (119 mM NaCl, 5 mM KCl, 5.6 mM glucose, 0.4 mM MgCl_2_, 25 mM PIPES, 40 mM NaOH, 1 mM CaCl_2_, and 0.1% bovine serum albumin; pH 7.2), and then incubated in 100 μL siraganian buffer supplemented with compound 48/80 (D-073, Sigma-Aldrich) or WEML for 1 h. Compound 48/80 was used as a positive control. The reaction was terminated via incubation at 4°C for 10 min. The fluorescence intensity of β-hexosaminidase in the supernatant was measured using a β-hexosaminidase activity assay kit (Cell Biolabs, San Diego, CA, USA) by using a 2104 EnVision Multilabel Plate Reader (PerkinElmer, Waltham, MA, USA) at an emission wavelength of 340 nm and an excitation wavelength of 450 nm. β-Hexosaminidase release (relative fluorescence units) was calculated by subtracting the fluorescence intensity of the control group from that of the experimental group.

### Statistical analysis

Unpaired two-tailed Student’s *t-*tests were used for statistical analysis of data. Values of *p* < 0.05 were considered significant.

## Results

### Activity of M. indica leaf extracts on expression of the hair growth-related genes

**Figure 1.**
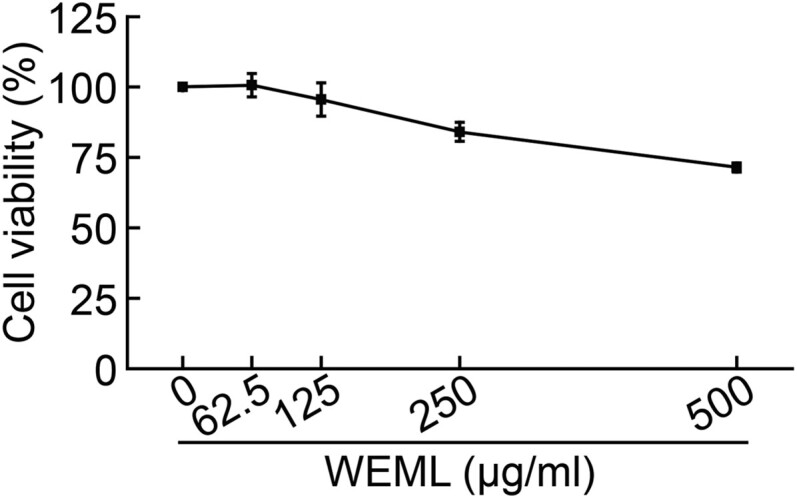
Effect of WEML on mitochondrial activity. HFDP cells were treated with indicated concentrations of WEML for 24 h, followed by an MTS assay. Results are expressed as the mean ± standard deviation (*n* = 3). WEML, water extract of *Mangifera indica* leaves.

**Figure 2.**
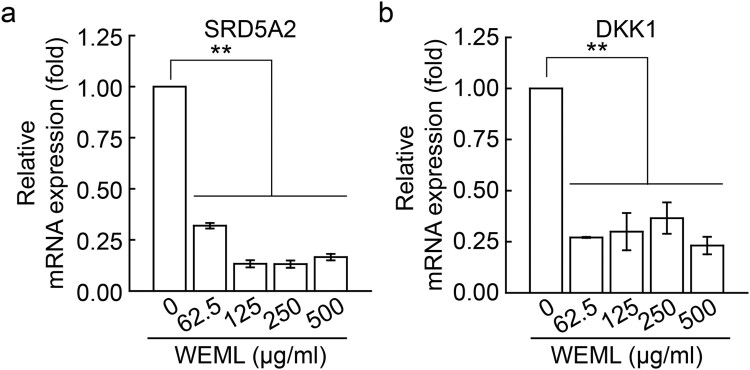
Effect of WEML on expression of hair loss-related genes. HFDP cells were treated with indicated concentrations of WEML for 36 h. Real-time reverse transcription-polymerase chain reaction was performed to analyze mRNA expression levels of the hair loss-related genes (A) SRD5A2 and (B) *DKK1*. Results are expressed as the mean ± standard deviation (*n* = 3). **p < 0.001. WEML, water extract of *Mangifera indica* leaves; SRD5A2, steroid 5-alpha reductase 2; *DKK1*, dickkopf-1.

To investigate the effect of WEML on cell viability, we first performed an MTS assay after treating HFDP cells with WEML at concentrations of 62.5–500 μg/ml for 24 h. WEML treatment did not significantly reduce the cell viability of cells to <75% (Figure 1). Although our previous study showed that *M. indica* leaves contain bioactive properties, their effects on hair growth, such as the inhibition of hair-related gene expression, are still unknown (Choi et al. 2018). In this study, HFDP cells, which are primary cells, were used to conduct experiments under conditions more similar to those of *in vivo*. We performed qRT-PCR to examine the regulatory effect of WEML on the mRNA expression levels of various genes related to hair growth. The inhibitory effect of some hair growth-related gene expression by WEML in DP cells was limited to SRD5A2 and *DKK1* genes in HFDP cells (Supplementary table 2), suggesting that the WEML treatment could downregulate SRD5A2 and *DKK1* (Figure 2).

### Expression level of Wnt target genes was increased after WEML treatment via downregulation of DKK1

**Figure 3.**
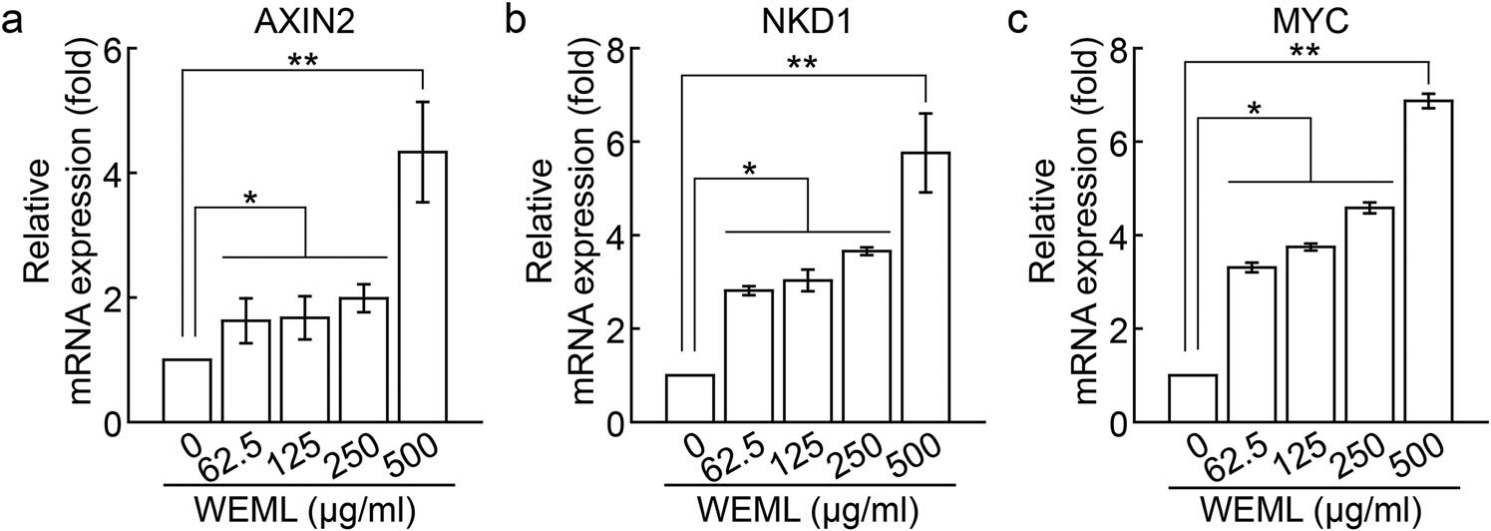
Effect of WEML on the expression of the Wnt target genes. HFDP cells were treated with indicated concentrations of WEML for 36 h. Real-time reverse transcription-polymerase chain reaction was performed to analyze mRNA expression levels of the Wnt target genes (A) *AXIN2*, (B) *NKD1*, and (C) *MYC*. Results are expressed as mean ± standard deviation (*n* = 3). *p < 0.05, **p < 0.01. WEML, water extract of *Mangifera indica* leaves; AXIN2, axin-2; NKD1, naked cuticle homolog 1.

**Figure 4.**
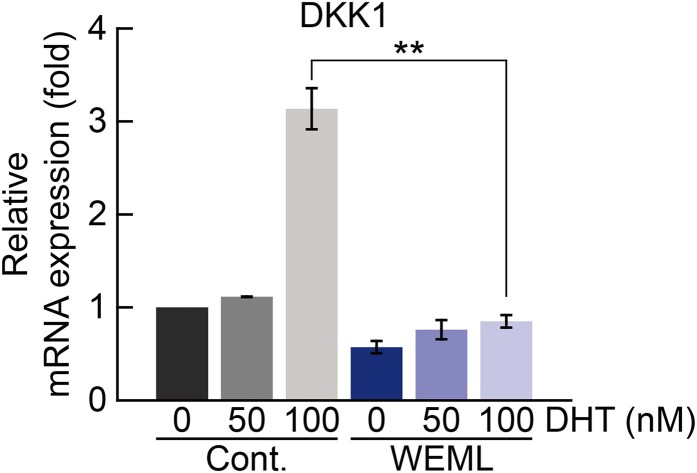
Effect of WEML on expression of the DHT-induced *DKK1*. HFDP cells were pretreated with WEML (250 μg/mL) for 36 h and then stimulated with indicated concentrations of DHT for 12 h. qRT-PCR was performed to analyze mRNA expression levels of *DKK1*. Results are expressed as the mean ± standard deviation (*n *= 3). **p < 0.01. WEML, water extract of *Mangifera indica* leaves; *DKK1*, dickkopf-1; DHT, dihydrotestosterone.

Based on the inhibition of *DKK1* expression via WEML treatment, we investigated the effect of WEML treatment on Wnt target gene expression. The mRNA expression levels of axin-2 (*AXIN2*), naked cuticle homolog 1 (*NKD1*), and *MYC* genes, which are well-known Wnt target genes, (Sick et al. 2006; Katoh and Katoh 2007) were increased in HFDP cells in a dose-dependent manner following WEML treatment (Figure 3). We confirmed that SRD5A2 expression level, which induces the conversion of testosterone to DHT, and that of *DKK1*, which plays a role in inhibiting Wnt signaling via the binding of DHT to AR, was reduced upon WEML treatment. Regarding the potential for WEML-induced hair growth, we investigated whether WEML treatment inhibited DHT conversion via downregulation of SRD5A2 to suppress *DKK1* expression, thereby inducing an increase in Wnt target gene expression. We found that in the absence of WEML treatment, *DKK1* was upregulated via DHT treatment, as expected; however, *DKK1* expression induced by DHT treatment was significantly decreased after WEML treatment (Figure 4). These results suggest that the increase in Wnt target gene expression through WEML treatment not only downregulates SRD5A2, may also negatively act downstream of DHT/AR that upregulates *DKK1*.

### WEML treatment did not induce allergic reactions

**Figure 5.**
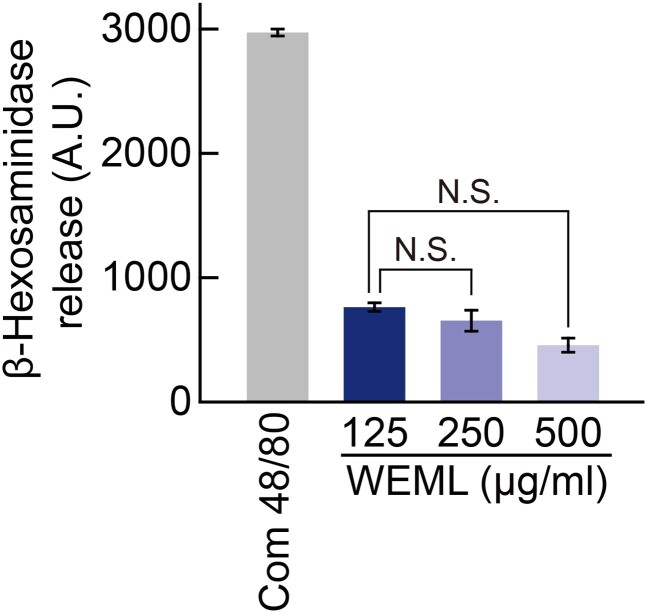
Effect of WEML on degranulation in RBL-2H3 cells. RBL-2H3 cells were treated with compound 48/80 (1 mg/mL) and indicated concentrations of WEML for 1 h. Compound 48/80 was used as a positive control for degranulation. Release of β-hexosaminidase was measured via fluorometric analysis. Results are expressed as the mean ± standard deviation (*n* = 3). N.S., no statistical significance. *p < 0.05. WEML, water extract of *Mangifera indica* leaves; AU, arbitrary units.

Since WEML treatment regulated the expression of hair growth-related genes, we expect that WEML treatment may enhance hair growth in an *in vivo* mouse model based on activity between DHT and AR as well as SRD5A2 function. Before verifying the effect on hair growth in the *in vivo* mouse model treated with WEML, we determined whether WEML treatment induced an allergic reaction in skin. To evaluate allergic reactions against WEML, the release of β-hexosaminidase was measured after treatment of RBL-2H3 cells with WEML. As a positive control, the cells were treated with compound 48/80, an oligomeric mixture of condensation products of N-methyl-p-methoxyphenethylamine and formaldehyde which is known to promote mast cell degranulation. WEML treatment at increasing concentrations did not induce the release of β-hexosaminidase (Figure 5). These results suggest that side effects due to direct allergic reactions may not occur.

### WEML treatment promoted hair growth in mice

**Figure 6.**
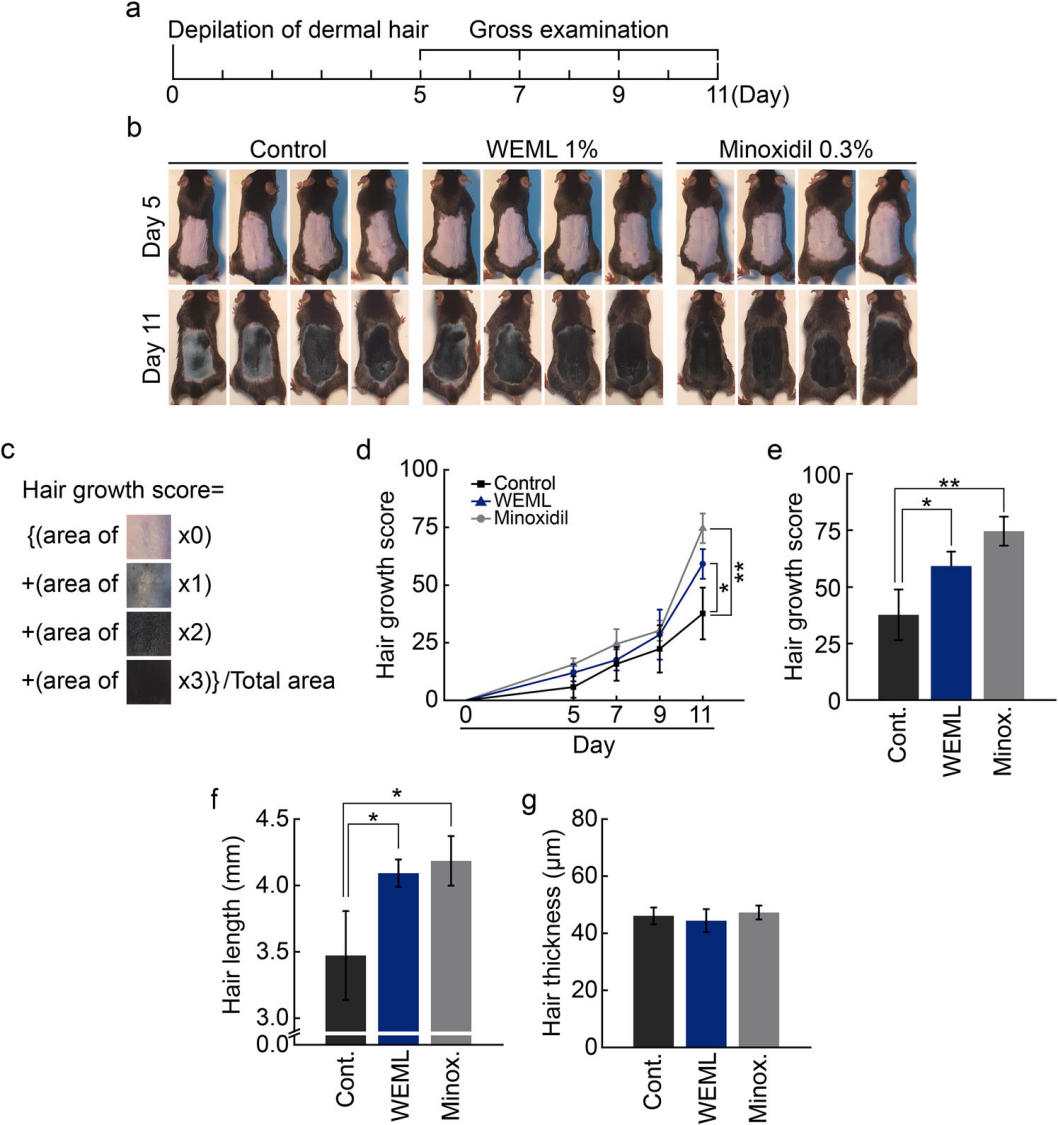
Effect of WEML on hair growth in C57BL/6J mice. (A) Schematic diagram for the procedure of the *in vivo* experiment. (B) WEML (1%) and minoxidil (0.3%) were topically applied on the dorsal skin for 11 d. (C) Image of hair growth index calculated with area ratio and scores. (D) Hair growth score was calculated during 5–11 d. (E) Hair growth score, (F) hair length, and (G) hair thickness were calculated during 11 d. *n* = 8 (biological replicates); data are expressed as the mean ± standard error of mean. *p < 0.05 and **p < 0.01. WEML, water extract of *Mangifera indica* leaves.

Next, we evaluated the effect of WEML application on hair growth in an *in vivo* mouse model. Melanocytes do not exist in the epidermis and only exist in the hair follicles in C57BL/6 mice with black body hair; since melanin synthesis in the hair follicles occurs only during the growth phase, the skin color turns black during the growth phase and pink during the resting and regression phases (Ermak and Slominski 1997; Deng et al. 2021). Therefore, since melanin synthesis correlates well with the hair growth cycle, the hair growth cycle can be evaluated based on skin color without skin biopsy, and is widely used in hair physiology research. In addition, when C57BL/6 mice reach 6–7 weeks of age, most of their hair is in the resting phase and their skin color turns pink. Therefore, to investigate the effect of WEML on hair growth based on a comparative experiment with minoxidil, each sample was applied after removing hair from the back of a 6-week-old C57BL/6 mouse model (Figure 6(A)). Minoxidil, and not finasteride, was applied as a positive control on the skin. In the case of minoxidil, if the total daily dose of 3% minoxidil available on the market exceeds 2 mL, then systemic effects related to vasodilation, such as fluid retention, hypotension, and tachycardia, may occur due to increased systemic absorption. When testing on mice with a smaller body, a 1/10 dilution with vehicle was used at a 0.3% concentration. When WEML is applied to the skin, it must penetrate the stratum corneum and epidermis to affect the dermis layer. Considering the difference in component permeability, WEML was applied at a higher concentration of 1% than when directly applied to cells. The pattern of hair growth was visually confirmed when applying each sample to the depilated back of mice from the day after hair removal; there was no significant difference in each test group until the 9th day. However, on the 11th day, it was confirmed that the rate of hair growth from hair follicles was higher in the minoxidil-treated and WEML-treated groups than that in the vehicle group (Figure 6(B–D)). There was no difference in hair thickness after minoxidil and WEML treatment compared to that in the vehicle-treated group; however, a difference in hair length was observed (Figure 6(E,F)).

## Discussion

**Figure 7.**
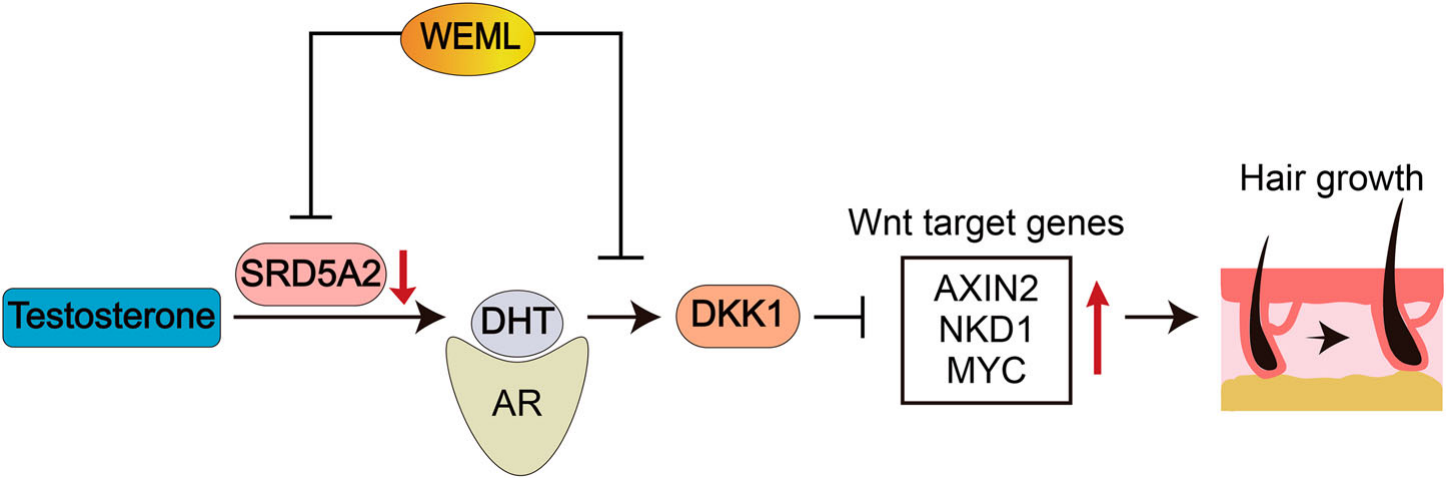
Schematic diagram of hair growth promotion according to activation of Wnt signaling pathway by WEML treatment. WEML treatment significantly reduced the expression levels of both *DKK1* and SRD5A2 involved in Wnt signal suppression activity and DHT synthesis, respectively, in HFDP cells. In addition, it effectively up-regulated Wnt target genes and down-regulated *DKK1* expression increased by DHT treatment.

In this study, we investigated the effect of WEML on hair growth. WEML showed hair growth-promoting activity in an *in vivo* mouse model, which was attributed to the downregulation of SRD5A2 and inhibition of Wnt signaling (Figure 7). This mechanism of WEML-mediated action is expected to enhance hair growth more effectively in human applications.

Recently, research on the development of inhibitors or therapeutic agents against hair loss for the development of drugs, quasi-drugs, and cosmetics is increasing (Madnani and Khan 2013). Most hair loss-related medicines are chemically synthesized products that have side effects, such as male gynecomastia, decreased ejaculation, decreased libido, hirsutism, dry scalp, and allergic contact dermatitis among other effects (Mermi et al. 2014). To minimize these side effects, the development of medicines derived from natural products, including herbal preparations with low toxicity, is being studied (Shen et al. 2018). At present, natural herbal medicines that enhance hair growth and prevent hair loss have been prepared from goji berries, eoseongcho, baeksuo, ginkgo biloba, burdock root, tangyak, chrysanthemum, chrysanthemum leaf, ginger, bay leaf, green tea, baekjiin, sagebrush, ginseng, dandelion, and orchids (Kobayashi et al. 1993; Chung et al. 2017). Various natural material extracts have been tested, such as ginseng, wild chrysanthemum, sangbaek, raw pine needles, cheonggung, and manhyeongja (Park et al. 2015; Shen et al. 2018). However, studies on the exact mechanism underlying enhancement of hair growth and inhibition of hair loss induced by treatment with these natural products are lacking.

The components of *M. indica* leaves include flavonoids, xylose, rosin, phenols, and constituents, such as glucose, catechin (VI), galactose, arabinose, rhamnose, estregole, leucine, valine, protocatechuic acid, alanine, glycine, kinic acid, tannins, α-pinene, β-pinene (VI), linalool, and limonene (Kumar et al. 2021). Among these components, anthocyanins have a strong antioxidant activity and remove free radicals that damage blood vessels, organs, and aging via various actions (Rocha Ribeiro et al. 2010). It has been reported that when these anthocyanins are continuously ingested, they show positive effects in the improvement of diabetic vasculopathy, diabetic retinopathy, and visual acuity via rhodopsin resynthesis. In addition, mango contains a large number of antioxidants, polyphenols, and carotenoids compared to those present in other fruits (Itoh et al. 2020; Mirza et al. 2021). Carotenoids are known to strengthen immunity and protect normal cells via antioxidant action to prevent diseases such as cancer, degenerative diseases, and aging; they also adsorb toxic substances present in the intestine and excrete them via bowel movement (Ngo et al. 2019; Mirza et al. 2021). Therefore, it is necessary to further investigate the various active ingredients present in *M. indica* leaves and identify components that promote hair growth by inhibiting the expression of SRD5A2 and upregulating *DKK1* caused by DHT.

Hormonal imbalance caused by various genetic and environmental factors leads to hair loss (Redler et al. 2017). In this study, we aimed to discover a new function of the active ingredient extract of *M. indica* leaves in the prevention and treatment of hair loss and to elucidate the underlying mechanism. Excessive production of DHT by SRD5A2, which is a representative cause of male pattern hair loss, induces the expression of *DKK1*, an inhibitor of the Wnt signaling pathway, via AR stimulation, which ultimately leads to the blockade of the Wnt signaling pathway. In turn, the blocked Wnt signaling inhibits hair growth via cell death (Ceruti et al. 2018). Therefore, inhibition of transcriptional expression of SRD5A2 may assist in the prevention and development of treatment for male pattern hair loss, and it is important to restore the activity of the Wnt signaling pathway suppressed by DHT (Inui and Itami 2013). The mRNA expression level of *DKK1* was decreased by WEML treatment, and when 100 nM of DHT was co-treated, the mRNA expression level of *DKK1* was decreased more significantly. This result may be due to the activity of DHT and AR as well as regulation of SRD5A2 in hair growth. Therefore, the regulation of SRD5A2 and the binding of DHT and AR act in a dual mode on the hair growth mechanism, how it affects the binding of DHT and AR has not yet been elucidated, further studies are required. The dual-mode action of *M. indica* leaf extracts in the suppression of SRD5A2 expression and restoration of the Wnt signaling pathway suppressed by DHT is important for overcoming the technical limitations of existing hair loss treatments and for treating male pattern hair loss.

## Supplementary Material

Supplemental Material

## Data Availability

The data that support the findings of this study are available from the corresponding author upon reasonable request.
