# Supplementary material for: Mangifera Indica leaf extracts promote hair growth via activation of Wnt signaling pathway in human dermal papilla cells
Source: Anim Cells Syst (Seoul). 2022 Jun 11;26(3):129–36. doi: 10.1080/19768354.2022.2085790 (PMC9246026; doi:10.1080/19768354.2022.2085790)
Supplement: Supplemental Material [file TACS_A_2085790_SM9975.zip › Supplementary table 1.docx]

Supplementary table 1. Primer information for qRT-PCR

| Genes |  | Sequence (5′-3′) |
| --- | --- | --- |
| DKK1 | Forward | TCT GGA ATA CCC ATC CAA GG |
|  | Reverse | ATG CGT CAC GCT ATG TGC T |
| SRD5A2 | Forward | AAG GAC TCC ATT TCC AGT GC |
|  | Reverse | ACG GTA CTT CTG GGC CTC TT |
| NKD1 | Forward | AGA AGA TGG AGA GAG TGA GC |
|  | Reverse | TGG ATG CTG GGG AGT GGT TGA |
| AXIN2 | Forward | TGT GAG GTC CAC GGA AAC TG |
|  | Reverse | CGT CAG CGC ATC ACT GGA TA |
| MYC | Forward | CGT AGT TGT GCT GAT GTG TGG |
|  | Reverse | CTC GGA TTC TCT GCT CTC CTC |
| β-actin | Forward | TCA CCC ACA CTG TGC CCA TCT ACG A |
|  | Reverse | CAG CGG AAC CGC TCA TTG CCA ATG |
| SGK | Forward | ATA CAA GAC AGC TCC CAG GC |
|  | Reverse | TCG GAC TCT GCA AGG AGA AC |
| EGR1 | Forward | GGA AAA GCG GCC AGT ATA GG |
|  | Reverse | AGC CCT ACG AGC ACC TGA C |
| SRD5A1 | Forward | CCA ACA GTG GCA TAG GCT TT |
|  | Reverse | CTA CCA GTA CGC CAG CGA GT |
