# Supplementary material for: Mangifera Indica leaf extracts promote hair growth via activation of Wnt signaling pathway in human dermal papilla cells
Source: Anim Cells Syst (Seoul). 2022 Jun 11;26(3):129–36. doi: 10.1080/19768354.2022.2085790 (PMC9246026; doi:10.1080/19768354.2022.2085790)
Supplement: Supplemental Material [file TACS_A_2085790_SM9975.zip › Supplementary table 2.docx]

Supplementary table 2. Effect of WEML on expression of hair loss-related genes

| Genes | WEML concentration (µg/ml) | Fold change |
| --- | --- | --- |
| DKK1 | 0 | 1.00 ± 0.0 |
|  | 67.5 | 0.27 ± 0.0 |
|  | 125 | 0.30 ± 0.1 |
|  | 250 | 0.37 ± 0.1 |
|  | 500 | 0.23 ± 0.0 |
| SRD5A2 | 0 | 1.00 ± 0.0 |
|  | 67.5 | 0.32 ± 0.0 |
|  | 125 | 0.13 ± 0.0 |
|  | 250 | 0.13 ± 0.0 |
|  | 500 | 0.17 ± 0.0 |
| SGK | 0 | 1.00 ± 0.0 |
|  | 67.5 | 2.24 ± 0.0 |
|  | 125 | 3.24 ± 0.0 |
|  | 250 | 3.50 ± 0.2 |
|  | 500 | 3.82 ± 0.2 |
| EGR1 | 0 | 1.00 ± 0.0 |
|  | 67.5 | 2.09 ± 0.1 |
|  | 125 | 2.69 ± 0.1 |
|  | 250 | 1.56 ± 0.0 |
|  | 500 | 1.59 ± 0.1 |
| SRD5A1 | 0 | 1.00 ± 0.0 |
|  | 67.5 | 1.61 ± 0.0 |
|  | 125 | 2.76 ± 0.4 |
|  | 250 | 3.47 ± 0.1 |
|  | 500 | 4.16 ± 0.1 |
